# Role of miR-222-3p in c-Src-Mediated Regulation of Osteoclastogenesis

**DOI:** 10.3390/ijms17020240

**Published:** 2016-02-16

**Authors:** Shinya Takigawa, Andy Chen, Qiaoqiao Wan, Sungsoo Na, Akihiro Sudo, Hiroki Yokota, Kazunori Hamamura

**Affiliations:** 1 Department of Biomedical Engineering, Indiana University—Purdue University Indianapolis, Indianapolis, IN 46202, USA; stakigaw@iupui.edu (S.T.); andychen@umail.iu.edu (A.C.); wanq@purdue.edu (Q.W.); sungna@iupui.edu (S.N.); hyokota@iupui.edu (H.Y.); 2 Department of Orthopaedic Surgery, Mie University Graduate School of Medicine, Mie 514-8507, Japan; a-sudou@clin.medic.mie-u.ac.jp; 3 Department of Pharmacology, School of Dentistry, Aichi-Gakuin University, Nagoya 464-8650, Japan

**Keywords:** microarray, miRNA, osteoclastogenesis, RAW264.7 cells, c-Src

## Abstract

MicroRNAs (miRNAs) are small non-coding RNAs that play a mostly post-transcriptional regulatory role in gene expression. Using RAW264.7 pre-osteoclast cells and genome-wide expression analysis, we identified a set of miRNAs that are involved in osteoclastogenesis. Based on *in silico* analysis, we specifically focused on miR-222-3p and evaluated its role in osteoclastogenesis. The results show that the inhibitor of miR-222-3p upregulated the mRNA levels of nuclear factor of activated T-cells, cytoplasmic 1 (NFATc1) and tartrate-resistant acid phosphatase (TRAP), while its mimicking agent downregulated their mRNA levels. Western blot analysis showed that its inhibitor increased the protein levels of TRAP and cathepsin K, while its mimicking agent decreased their levels. Genome-wide mRNA expression analysis in the presence and absence of receptor activator of nuclear factor κ-B ligand (RANKL) predicted c-Src as a potential regulatory target of miR-222-3p. Live cell imaging using a fluorescence resonance energy transfer (FRET) technique revealed that miR-222-3p acted as an inhibitor of c-Src activity, and a partial silencing of c-Src suppressed RANKL-induced expression of TRAP and cathepsin K, as well as the number of multi-nucleated osteoclasts and their pit formation. Collectively, the study herein demonstrates that miR-222-3p serves as an inhibitor of osteoclastogenesis and c-Src mediates its inhibition of cathepsin K and TRAP.

## 1. Introduction

MicroRNAs (miRNAs) are small non-coding RNA molecules that participate in various gene regulatory processes. They may reorganize chromatin and silence specific genes, cleave and destabilize mRNAs, or inhibit transcriptional initiation and elongation [1]. Chronic lymphocytic leukemia is the first human disease that was shown to be associated with miRNA [2], and since then involvement of miRNAs in tumor growth and metastasis has been reported [2,3]. The regulatory role of miRNAs has also been identified in homeostasis of organ systems such as the cardiovascular system, the nervous system, and musculoskeletal system [4,5,6], as well as in the maintenance and development of stem cells [7].

The specific aim of this study is to evaluate a potential role of miRNAs in osteoclastogenesis. Osteoclastogenesis is a developmental process of pre-osteoclast cells in the hematopoietic lineage. In the self-fusion process to form mature multinucleated cells, receptor activator of nuclear factor κ-B ligand (RANKL) acts as a potent stimulator and activates nuclear factor κ-B (NFκB) signaling and a transcription factor, nuclear factor of activated T-cells, cytoplasmic 1 (NFATc1) [8]. In the response to RANKL’s stimulation, NFATc1 is considered to serve as a master transcription factor, and other regulatory factors such as macrophage colony-stimulating factor (M-CSF), c-Fos, PU.1, and TRAF6 are also known to be activated [8]. It is reported that miRNAs such as miR-34a, miR-99b, miR-223, miR-365, miR-378, and miR-451 are involved in osteoclastogenesis [9,10,11,12,13,14]. However, little is known about the regulatory mechanism of their actions and the genes directly regulated by these miRNAs.

In this study, we conducted genome-wide expression profiling of both miRNAs and mRNAs using RAW264.7 pre-osteoclast cells, followed by *in silico* data analysis, primarily using principal component analysis. Expression analysis led us to focus on miR-222-3p, which presented a consistent expression profile during osteoclastogenesis. In the presence and absence of miR-222-3p’s inhibitor and mimicking agent, its influence on the selected osteoclast marker genes such as TRAP and cathepsin K was examined. Furthermore, miR-222-3p’s target genes were predicted using publicly available software tools, and the predicted regulatory pathway was evaluated using inhibitor and mimicking agents, as well as siRNA specific to miR-222-3p’s target gene and FRET (fluorescence resonance energy transfer)-based live cell imaging.

## 2. Results

### 2.1. Receptor Activator of Nuclear Factor κ-B Ligand (RANKL) Responsive miRNAs

**Figure 1:**
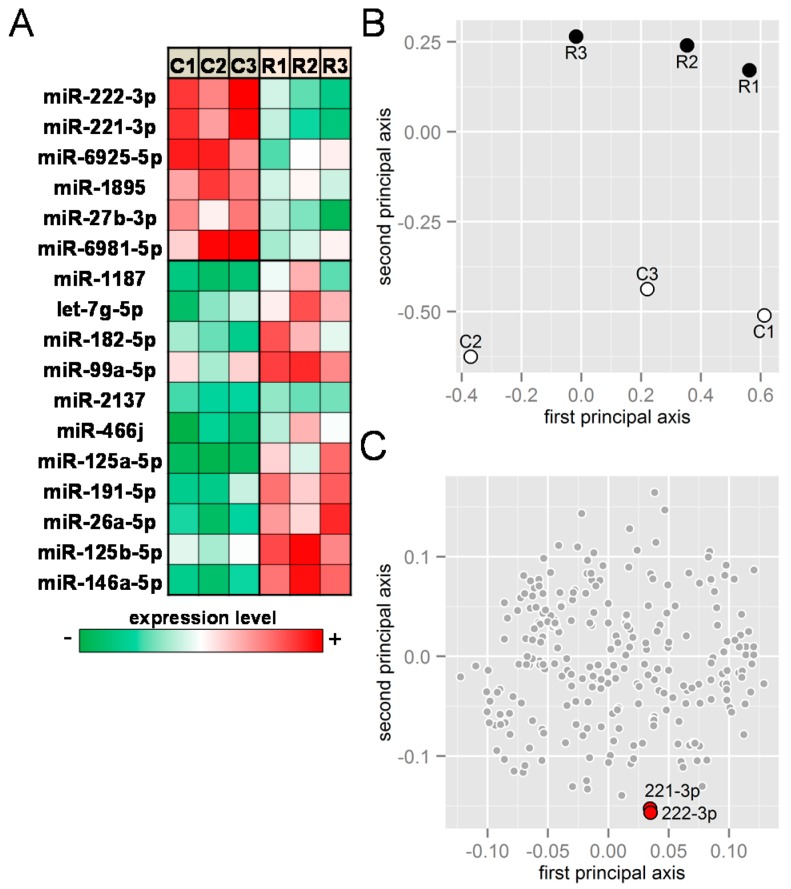
Microarray-based miRNA expression analysis. Cells were stimulated with 20 ng/mL RANKL for two days. (**A**) Heat map of the selected miRNAs (signal values < 500 and *p* < 0.05) in response to receptor activator of nuclear factor κ-B ligand (RANKL). The green and red colors indicate downregulation and upregulation, respectively. Of note, C1, C2, and C3 are control samples, and R1, R2, and R3 are RANKL-treated samples; (**B**) Principal component analysis (PCA) for six samples (C1–C3, and R1–R3) in the first and second principal plane; and (**C**) PCA for miRNAs in the first and second principal plane. Two miRNAs (221-3p and 222-3) are positioned with the smallest values along the second principal axis.

In response to RANKL treatment for two days, expression levels of ~20 miRNAs were significantly altered in the microarray-based assay (Figure 1). In particular, the heat map of the selected miRNAs (signal value > 500 and *p* < 0.05) shows that miRNAs such as miR-221-3p and miR-222-3p were downregulated by RANKL, while miRNAs such as miR-125b-5p and miR-182-5p were upregulated (Figure 1A). Principal component analysis (PCA), a statistical technique for finding representative axes in multidimensional data, was used to examine six miRNA samples (C1–C3, and R1–R3) to reveal that three RANKL-treated samples had a larger value in the second principal axis than three control samples (Figure 1B). Among 17 miRNAs in Figure 1A that significantly altered their expression levels by RANKL with high signal values (max signal > 500), two miRNAs (miR-221-3p and miR-222-3p) were positioned with the smallest second principal component values (Figure 1C).

### 2.2. PCR-Based mRNA and miRNA Expression Levels

**Figure 2:**
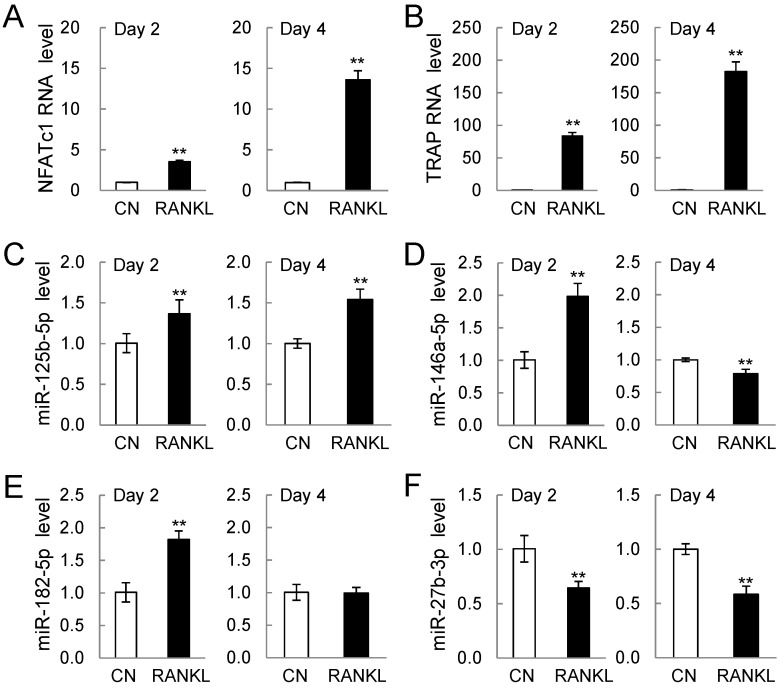

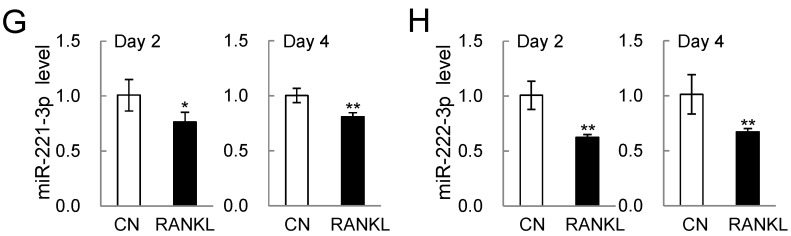
PCR-based mRNA and miRNA expression levels on Days 2 and 4 after RANKL addition (*n* = 5). CN = control sample; and RANKL = RANKL-treated sample (20 ng/mL): The single and double asterisks indicate *p* < 0.05 and *p* < 0.01, respectively. (**A**) nuclear factor of activated T-cells, cytoplasmic 1 (NFATc1) mRNA level; (**B**) tartrate-resistant acid phosphatase (TRAP) mRNA level; (**C**) miR-125b-5b level; (**D**) miR-146a-5p level; (**E**) miR-182-5p level; (**F**) miR-27b-3p level; (**G**) miR-221-3p level; and (**H**) miR-222-3p level.

The expression levels of miRNA, initially determined with microarrays, were re-evaluated using qPCR (Figure 2). Consistent with the stimulation of osteoclastogenesis by RANKL treatment, the mRNA levels of NFATc1 and TRAP were elevated on Days 2 and 4 (Figure 2A,B). The expression levels of three miRNAs (miR-125b-5p, miR-146a-5p, and miR-182-5p) in Figure 1A were elevated by RANKL treatment on Day 2, but their expression levels on Day 4 were either up, down, or unchanged (Figure 2C–E). Consistency on Days 2 and 4 were observed for three miRNA (miR-27b-3p, miR-221-3p, and miR-222-3p), which were continually downregulated by RANKL treatment (Figure 2F–H).

### 2.3. Effects of Inhibitor and Mimic miR221-3p and miR222-3p

**Figure 3:**
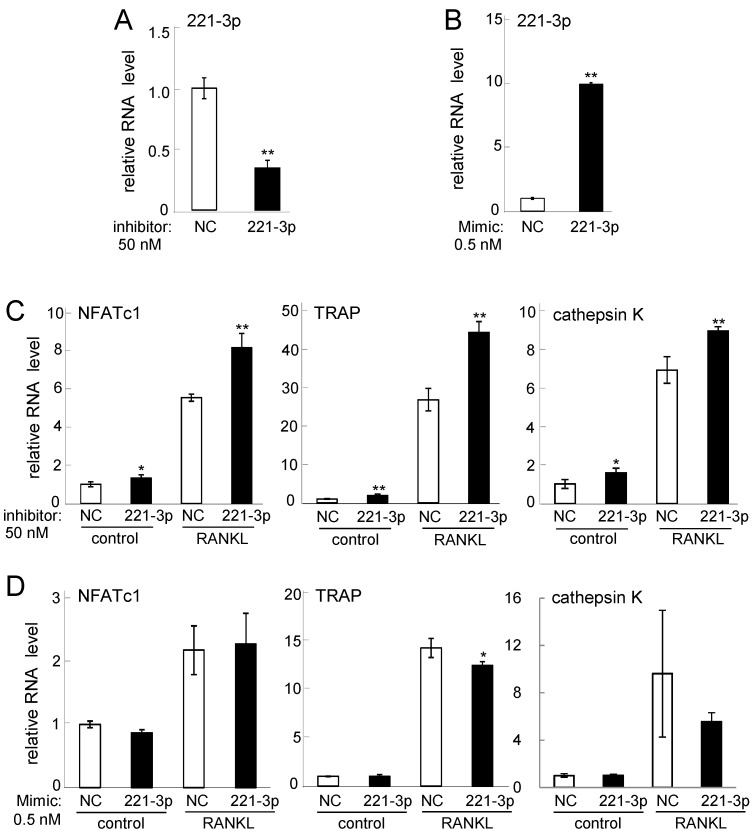
Effects of miR221-3p inhibitor and mimicking agent. Of note, 50 ng/mL RANKL was used for one day in (**C**,**D**). The sample number was three in (**A**,**B**), four in (**C**), and six in (**D**). NC = non-specific control. The single and double asterisks indicate *p* < 0.05 and *p* < 0.01, respectively. (**A**) miR-221-3p level in response to its inhibitor; (**B**) miR-221-3p level in response to its mimicking agent; (**C**) NFATc1, TRAP, and cathepsin K mRNA levels in response to miR-221-3p inhibitor; and (**D**) NFATc1, TRAP, and cathepsin K mRNA levels in response to miR-221-3p mimic.

**Figure 4:**
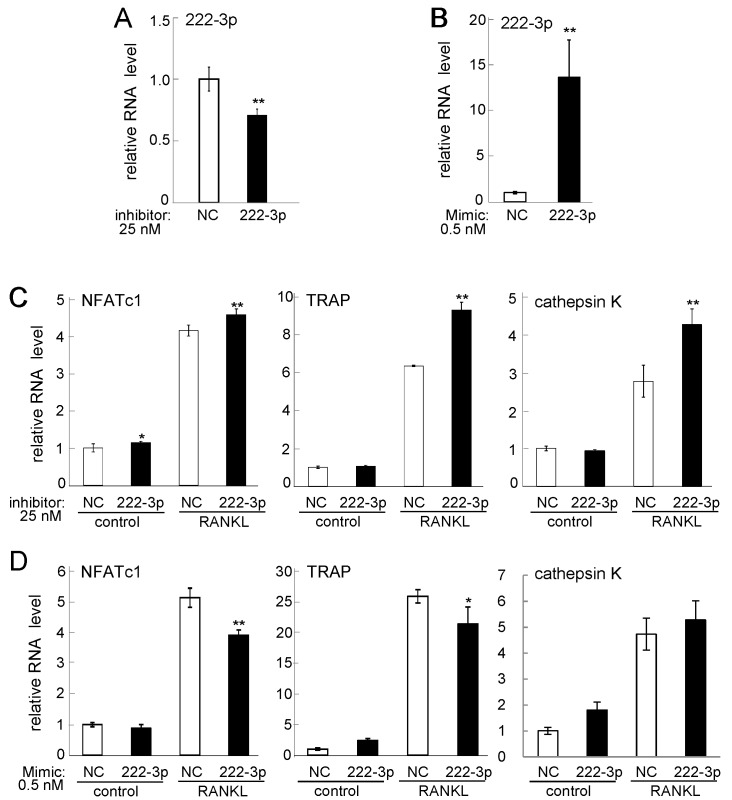
Effects of miR222-3p inhibitor and mimicking agent. Of note, 50 ng/mL RANKL was used for one day in (**C**,**D**). The sample number was three in (**A**,**B**), and four in (**C**,**D**). NC = non-specific control. The single and double asterisks indicate *p* < 0.05 and *p* < 0.01, respectively. (**A**) miR-222-3p level in response to its inhibitor; (**B**) miR-222-3p level in response to its mimicking agent; (**C**) NFATc1, TRAP, and cathepsin K mRNA levels in response to miR-222-3p inhibitor; and (**D**) NFATc1, TRAP, and cathepsin K mRNA levels in response to miR-222-3p mimic.

Since RANKL-driven downregulation of miR-221-3p and miR-222-3p have not been reported, we further evaluated their potential roles in osteoclastogenesis using their inhibitors and mimicking agents. In response to the inhibitor of miR-221-3p, the levels of NFATc1 mRNA, TRAP mRNA, and cathepsin K mRNA were significantly elevated (Figure 3A,C). Consistently, the mimicking agent reduced the mRNA level of TRAP, although the levels of NFATc1 mRNA and cathepsin K mRNA were not significantly changed (Figure 3B,D). We then examined the role of miR-222-3p using its inhibitor and mimicking agent (Figure 4). The mRNA levels of NFATc1, TRAP, and cathepsin K were upregulated by its inhibitor. Furthermore, the mRNA levels of NFATc1 and TRAP were downregulated by its mimicking agent (Figure 4A–D). Hereafter, the regulatory analysis was mainly conducted focusing on miR-222-3p.

### 2.4. Western Blot Analysis of Nuclear Factor of Activated T-Cells, Cytoplasmic 1 (NFATc1), Tartrate-Resistant Acid Phosphatase (TRAP), and Cathepsin K

**Figure 5:**
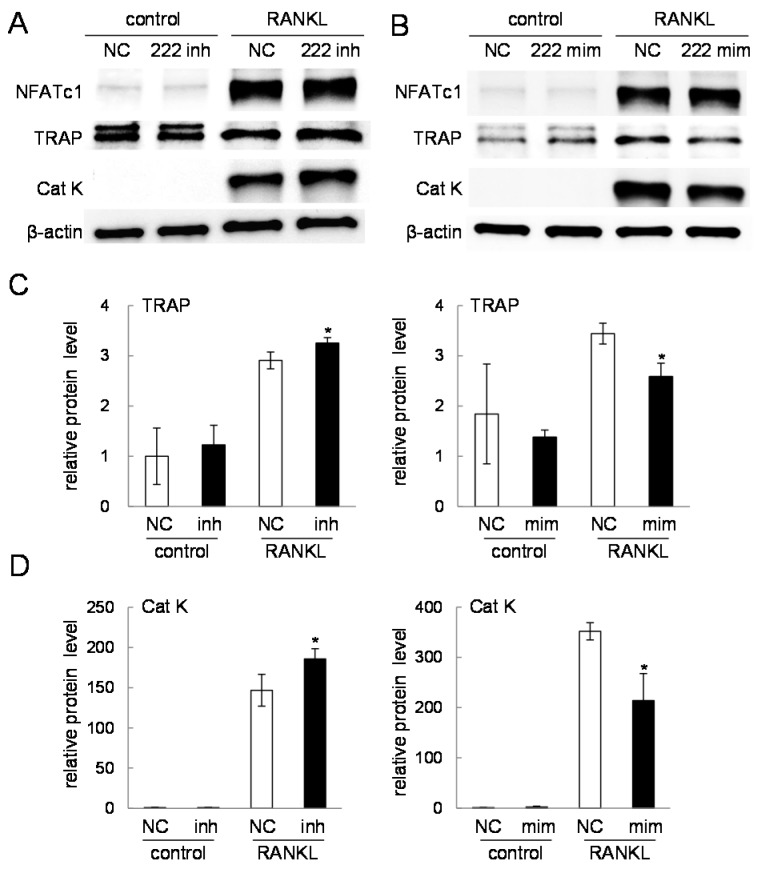
Western blot analysis of NFATc1, TRAP, and cathepsin K in response to the inhibitor or mimic specific to miR-222-3p. Of note, 50 ng/mL RANKL was used for three days in all cases. The sample number was three in (**C**,**D**). NC = non-specific control; inh = inhibitor; and mim = mimicking agent. The single asterisk indicates *p* < 0.05. (**A**) Expression of NFATc1, TRAP, and cathepsin K in response to the inhibitor of miR-222-3p; (**B**) Expression of NFATc1, TRAP, and cathepsin K in response to the mimic of miR-222-3p; and (**C**,**D**) Quantified expression levels of TRAP and cathepsin K, respectively, in response to the inhibitor and mimic of miR-222-3p.

The mRNA expression analysis of NFATc1 and TRAP predicted that miR-222-3p may act as an inhibitor of osteoclastogenesis. To further examine the role of miR-222-3p in osteoclast development, we determined the protein expression of NFATc1, TRAP, and cathepsin K in the presence of RANKL. In response to its inhibitor, the protein levels of TRAP and cathepsin K were increased (Figure 5A). Furthermore, its mimicking agent decreased the protein levels of TRAP and cathepsin K (Figure 5B). However, the level of NFATc1 protein was not altered in response to the inhibitor or mimicking agent.

### 2.5. RANKL Responsive Genes and the Prediction of miRNA Responsive Genes

**Figure 6:**
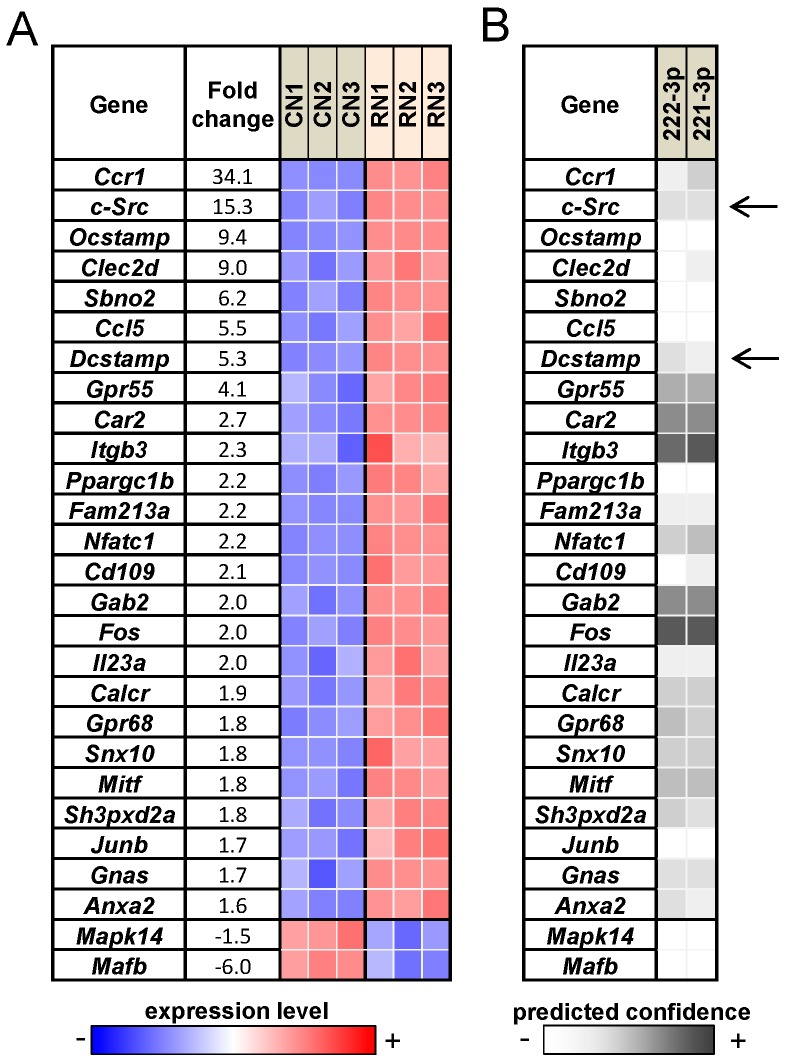
RANKL responsive genes and their potential link to miRNAs. (**A**) Heat map of RANKL-responsive genes that are related to osteoclasts. The blue and red colors indicate downregulation and upregulation, respectively. Note that CN1, CN2, and CN3 are control samples, and RN1, RN2, and RN3 are RANKL-treated samples; and (**B**) Predicted link between RANKL-responsive genes and miRNAs. The grey code indicates a confidence measure of *in silico* prediction. The arrows indicates they were chosen for further study.

To predict potential target genes that are regulated by RANKL-responsive miRNAs, we conducted genome-wide mRNA analysis followed by *in silico* prediction. First, we selected 27 osteoclast-linked genes whose mRNA levels were altered more than 1.5-fold by RANKL treatment (Figure 6A). These genes are ordered according to the fold change in their mRNA expression levels in response to RANKL treatment. We then predicted whether those 27 genes were potentially downregulated by RANKL-responsive miRNAs 221-3p and 222-3p (Figure 6B). The target prediction algorithms revealed several potential targets of 222-3p (*Ccr1*, *c-Src*, *Dcstamp*, *Gpr55*, *Car2*, *Itgb3*, *etc.*). Given their relatively high fold-changes as well as confidence in prediction, c-Src and Dcstamp were chosen for further study.

### 2.6. c-Src Expression and Activity in Response to the Inhibitor of miR-222-3p

**Figure 7:**
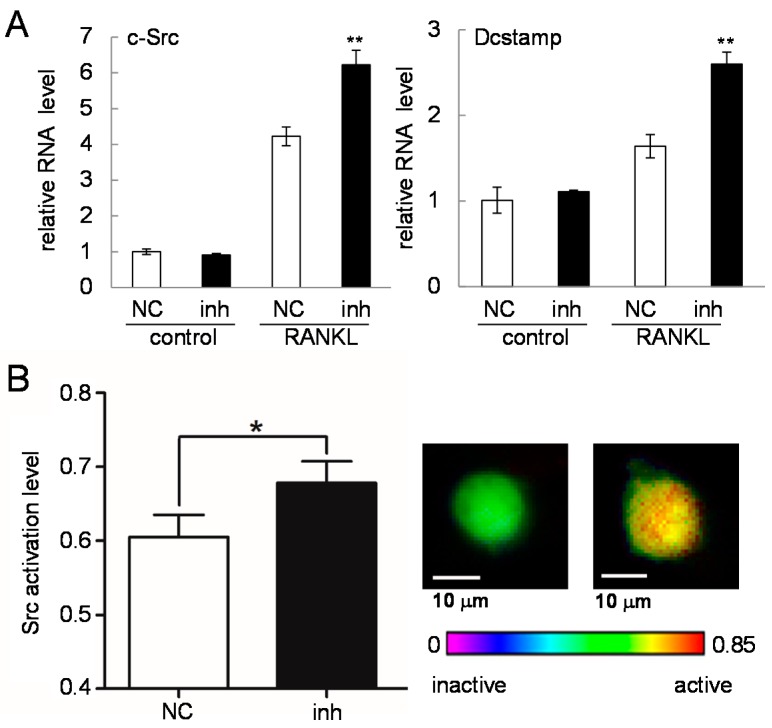
Responses of c-Src and Dcstamp to the inhibitor of miR-222-3p. Of note, 50 ng/mL RANKL was used for one day. The sample number was four in (**A**), and the scale bar is 10 µm in (**B**). NC = non-specific control; and inh = inhibitor of miR-222-3p. The single and double asterisks indicate *p* < 0.05 and *p* < 0.01, respectively. (**A**) c-Src and Dcstamp mRNA levels in response to the inhibitor of miR-222-3p; and (**B**) c-Src activity levels in response to the inhibitor of miR-222-3p.

Since c-Src and Dcstamp were highly responsive to RANKL treatment and predicted to be linked to miR-222-3p, we determined the mRNA levels of these two genes in the presence and absence of the inhibitor of miR-222-3p. Consistent with the prediction by *in silico* analysis, the result showed that their mRNA levels were elevated in response to miR-222-3p’s inhibitor agent (Figure 7A). To further test the involvement of miR-222-3p, the cells were co-transfected with a FRET-based c-Src biosensor and the inhibitor of miR-222-3p. The data revealed that, 24 h post-transfection, c-Src activity was elevated by the inhibitory agent (Figure 7B).

### 2.7. Effects of Silencing c-Src in RANKL-Driven Osteoclastogenesis

**Figure 8:**
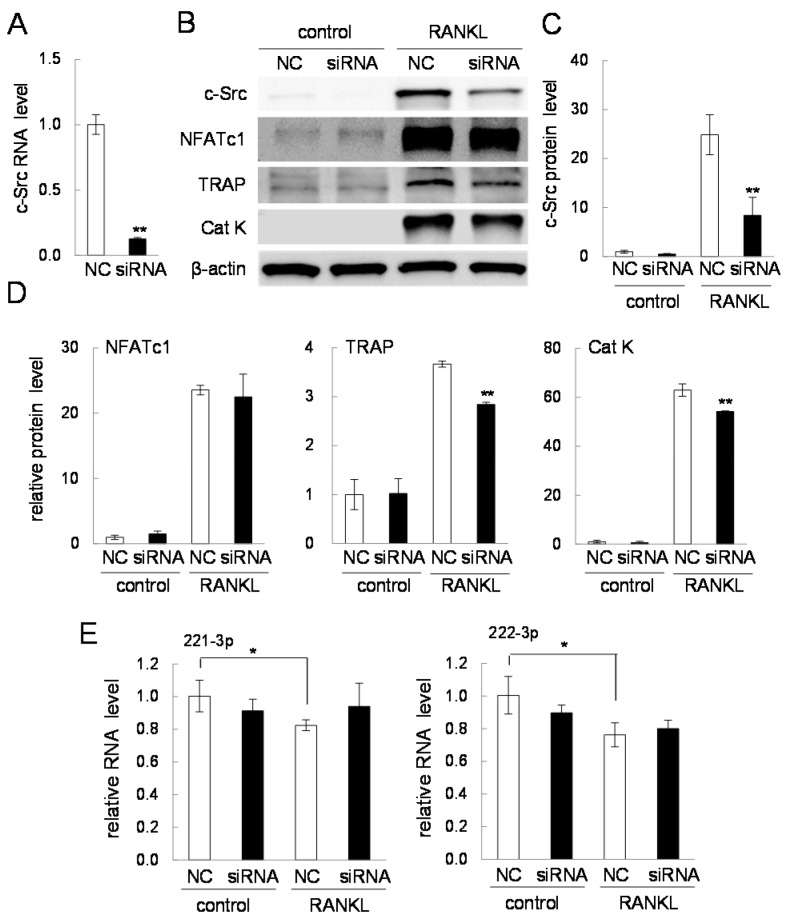
Effects of c-Src siRNA. Of note, 50 ng/mL RANKL was used for two days in (**B**–**E**). The sample number was three in all cases. NC = non-specific control siRNA. The single and double asterisks indicate *p* < 0.05 and *p* < 0.01, respectively. (**A**) c-Src mRNA levels in response to c-Src siRNA; (**B**) Western blot analysis of c-Src, NFATc1, TRAP, and cathepsin K in response to c-Src siRNA; (**C**) Quantified protein levels of c-Src in response to c-Src siRNA; (**D**) Quantified protein levels of NFATc1, TRAP and cathepsin K in response to c-Src siRNA; and (**E**) Levels of miR-221-3p and miR-222-3p in response to c-Src siRNA.

To evaluate the role of c-Src in RANKL-driven osteoclastogenesis, we employed siRNA specific to c-Src and determined the protein levels of NFATc1, TRAP, and cathepsin K. The significant reduction in the mRNA and protein levels of c-Src was confirmed (Figure 8A–C). The result with c-Src siRNA showed that the protein level of NFATc1 was not significantly altered. However, in response to RANKL treatment, the protein levels of TRAP and cathepsin K were significantly reduced by c-Src siRNA (Figure 8B,D). Of note, the expression of miR-221-3p or miR-222-3p was not significantly affected by c-Src siRNA treatment (Figure 8E).

**Figure 9:**
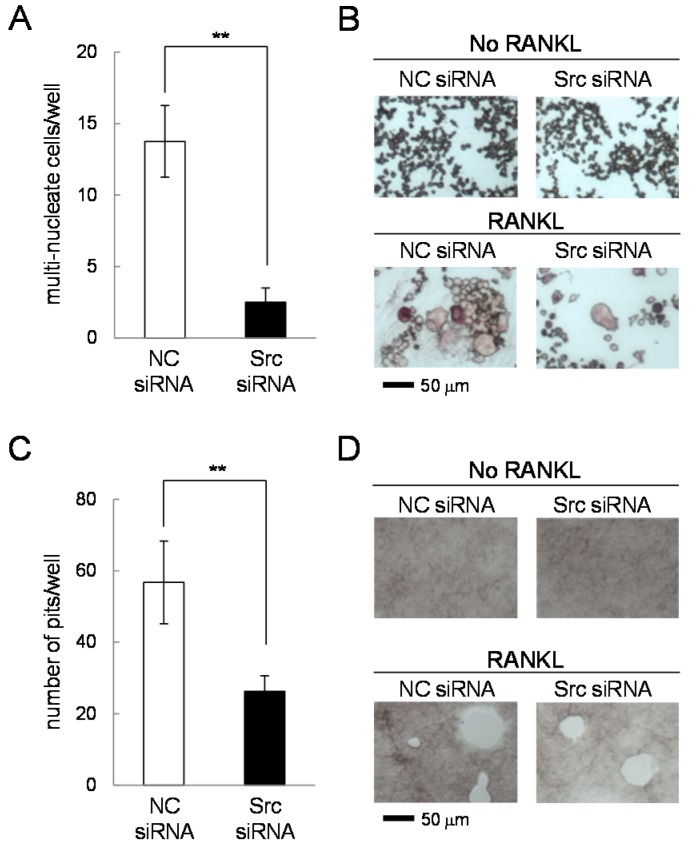
TRAP staining and osteoclast activity in the presence of c-Src siRNA. NC = non-specific control siRNA. The double asterisk indicates *p* < 0.01. (**A**,**B**) Number of multi-nucleated cells and TRAP staining in the presence and absence of 50 ng/mL RANKL for four days; and (**C**,**D**) Number of pits and their images in the presence and absence of 50 ng/mL RANKL for five days.

TRAP staining revealed that the number of multi-nucleated osteoclasts was significantly reduced in the presence of c-Src siRNA (Figure 9A,B). Furthermore, an osteoclast activity assay showed that the number of pits, formed by active osteoclasts, was lowered by c-Src siRNA treatment (Figure 9C,D).

## 3. Discussion

This study revealed that miR-222-3p acts as an inhibitory regulator of RANKL-driven osteoclastogenesis in RAW264.7 pre-osteoclast cells. Through genome-wide miRNA and mRNA expression analysis together with *in silico* predictions, miR-221-3p and miR-222-3p were highlighted as regulatory candidates. These two miRNAs were positioned at the corner in the PCA plane, indicating that their predicted inhibitory actions were strongest among RANKL responsive miRNAs. The result with qPCR revealed that the inhibitors of miR221-3p and miR-222-3p upregulated the mRNA levels of NFATc1 and TRAP. The mimicking agent of miR-222-3p downregulated the mRNA levels of NFATc1 and TRAP, while that of miR-221-3p did not significantly alter the mRNA level of NFATc1. Consistent with mRNA analysis, the inhibitor of miR-222-3p increased the protein levels of TRAP and cathepsin K, while its mimicking agent decreased these protein levels. Collectively, RNA-based loss-of-function and gain-of-function assays supported the inhibitory role of miR-222-3p in RANKL-driven upregulation of TRAP and cathepsin K.

In order to determine a signaling mechanism directly driven by miR-222-3p, 27 RANKL-responsive genes were selected as potential targets of miR-222-3p. *In silico* prediction using publicly available algorithms in miRWalk2.0 [15] assigned a confidence measure to those genes. Two genes, which were upregulated more than five-fold by RANKL and assigned a positive confidence measure in *in silico* prediction, were c-Src and Dcstamp. According to RNAhybrid software [16], a pairing of miR-222-3p (21 nucleotide long) and the 3′-end of c-Src’s untranslated region (UTR) yields the minimum free energy of −26 kcal/mol with 18 matches and three mismatches. In the response to miR-222-3p’s inhibitor and mimicking agent, we confirmed that the upregulation of c-Src mRNA and Dcstamp mRNA by RANKL was mediated by miR-222-3p.

**Figure 10:**
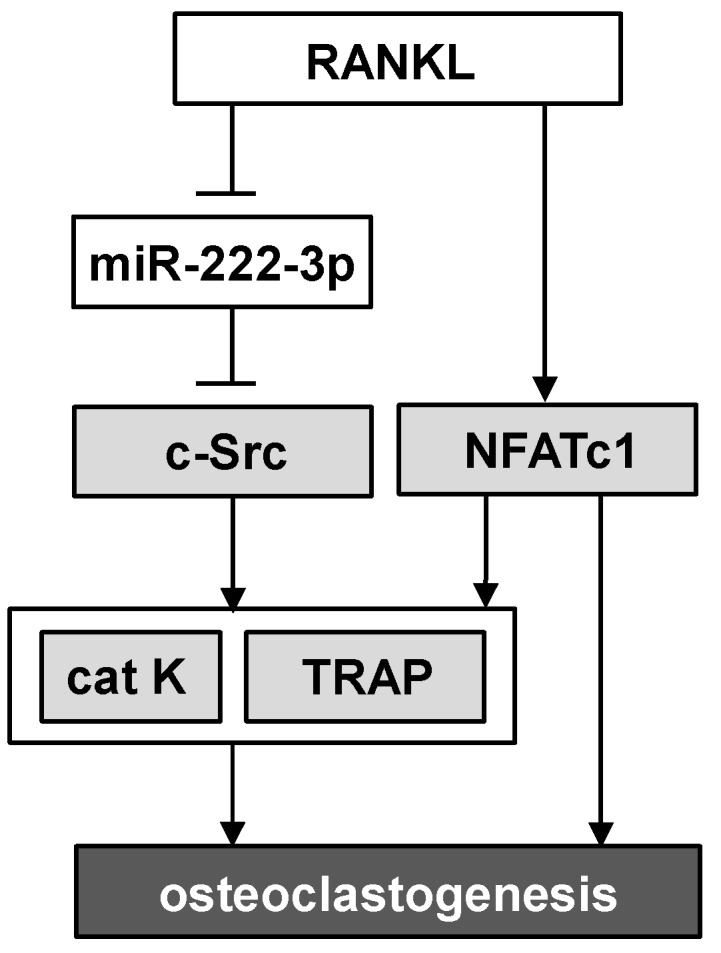
Schematic illustration of the proposed role of miR-222-3p in RANKL-stimulated osteoclastogenesis. Of note, cat K = cathepsin K.

Src family kinases are non-receptor tyrosine kinases, and c-Src is its proto-oncogene that promotes cellular survival, proliferation, and migration. It is reported that deletion of c-Src impairs bone resorbing activities by osteoclasts [17,18,19]. Histological analysis using TRAP staining as well as the osteoclast activity assay using pit formation revealed that a partial silencing of c-Src by RNA interference reduced the number of multi-nucleated osteoclasts and suppressed bone-resorbing activity. Using FRET-based live cell imaging, we determined the activity of c-Src in the presence and absence of miR-222-3p’s inhibitor agent. The FRET result showed that the baseline activity of c-Src was increased by the inhibitor agent. Furthermore, a partial silencing of c-Src by its siRNA suppressed miR-222-3p-driven downregulation of cathepsin K and TRAP. Dcstamp is also known to play a critical role in cell-cell fusion in osteoclasts [20], and miR-222-3p is involved in its regulation. Collectively, the proposed regulatory mechanism of miR-222-3p’s action is illustrated (Figure 10). Our qPCR results support the notion that miR-222-3p is inhibitory to c-Src that does not directly alter expression of miR-222-3p. Of note, while the mRNA level of NFATc1 was significantly downregulated by miR-222-3p, a reduction in its protein level was not significant.

Although the involvement of c-Src in miR-222-3p-driven regulation of osteoclastogenesis was predicted and validated, the rate of false positive in the *in silico* prediction turned out to be high. For example, a transcription factor, Fos, was predicted by every algorithm to be a target gene of miR-222-3p, but its expression was unchanged by the stimulation and inhibition of miR-222-3p (data not shown). A variety of prediction algorithms employ a combination of sequence matching, evolutionary conservation, adjacent nucleotide composition, sequence location, and the secondary structure of the targeted region [21], in which different weights and assumptions result in variations in predictions. It appears that most of these algorithms do not effectively take into account biological context such as dependence on cell types and physiological states [22].

In tumor proliferation and invasion, miRNAs play critical roles [23]. In various cancer types including colon, pancreas, prostate, and thyroid, the expression level of miR-222-3p is reported to be altered [3,24,25,26]. It is to be shown whether the observed modulation of miR-222-3p in tumor cells is induced by specific DNA mutations, and whether its modulation might be linked to metastasis to bone followed by osteolytic responses by osteoclasts.

Based on *in slico* predictions, we focused on miR-221-3p and miR-222-3p as two potential candidates for osteoclast regulation. These two miRNAs are known to form a cluster and regulate various genes [27], but it is also reported that they can act independently. For instance, miR-222 but not miR-221 is involved in neovascularization by regulating signal transducer and activator of transcription 5A (STAT5A) [28]. Hydroxyurea, which is used for the treatment of neoplastic diseases, elevates expression of miR-221 but decreases that of miR-222 [29]. Furthermore, the inhibition of miR-221 but not miR-222 suppresses a release of IL6 in patients with severe asthma [30].

Of note, the potential differences may exist in the role of miRNAs between RAW264.7 cells used in this study and primary cells such as bone marrow-derived cells. In summary, this study demonstrated an inhibitory role of miR-222-3p in RANKL-driven osteoclastogenesis. Genome-wide miRNA and mRNA analysis predicted c-Src as one of the miR-222-3p’s target genes. A partial silencing of c-Src using RNA interference revealed that miR-222-3p suppressed expression of the osteoclast marker genes such as TRAP and cathepsin K via c-Src. In bone-related metabolic diseases and cancer, the role of miRNAs has not been fully understood. By identifying miR-222-3p as a regulator of bone-resorbing osteoclasts, this study indicates a novel therapeutic direction in osteoporosis as well as osteolytic lesion in bone metastasis.

## 4. Experimental Section

### 4.1. Cell Culture

RAW264.7 pre-osteoclast cells (mouse monocytes/macrophages) were cultured in αMEM containing 10% fetal bovine serum and antibiotics (50 units/mL penicillin, and 50 μg/mL streptomycin; Life Technologies, Grand Island, NY, USA). Cells were cultured at 37 °C and 5% CO_2_ in a humidified incubator. To induce differentiation, cells were treated with 50 ng/mL RANKL (PeproTech, Rocky Hills, NC, USA) [31].

### 4.2. Microarray Experiments for Genome-Wide miRNAs and mRNAs

Genome-wide miRNA and mRNA expression analyses were conducted using µParaflo^®^ High Performance Microfluidic Microarrays (LC Sciences, Houston, TX, USA) and Mouse Gene 2.0 ST arrays (Affymetrix, Santa Clara, CA, USA), respectively. RAW264.7 cells were harvested 48 h after incubation with and without RANKL, and miRNA was isolated with a miRNeasy mini kit (Qiagen, Germantown, MD, USA). The samples for miRNA were labeled as C1–C3 (control) and R1–R3 (RANKL-treated), while the samples for mRNA were CN1–CN3 (control) and RN1–RN3 (RANKL-treated). Signal values for miRNA were background subtracted and normalized using the locally weighted scatterplot smoothing (LOWESS) method, while signal values for mRNA were normalized using robust multiarray average (RMA). The data analyzed in this study have been deposited in NCBI’s Gene Expression Omnibus [32]. The GEO Series accession number is GSE74847 (http://www.ncbi.nlm.nih.gov/geo/query/acc.cgi?acc=GSE74847). The heatmaps were generated for differentially expressed miRNAs and mRNAs at *p* < 0.05.

### 4.3. Principal Component Analysis

After pre-processing raw expression data, principal component analysis (PCA) was conducted for miRNAs using singular value decomposition, as outlined previously [33]. A set of highly expressed miRNAs whose minimum signal values were greater than 100 were selected, and principal component axes were defined. The six control and RANKL-treated samples (C1–C3 and R1–R3, respectively) as well as highly expressed miRNAs were mapped on the first and second principal component plane.

### 4.4. In Silico Prediction of miRNA Target Genes

In predicting miRNA target genes, we focused on miRNAs that were differentially expressed between the RANKL and control samples (*p* < 0.05) with a maximum signal value above 100. First, potential target genes were pre-selected, using the 3′-end of each gene’s untranslated sequence using 10 different prediction algorithms (miRWalk [15], Microt4 [34], miRanda [35], miRDB [36], miRMap [37], miRNAMap [38], PITA [39], RNA22 [40], RNAhybrid [16], and Targetscan [41]); Second, from the above potential genes, we further chose the genes that were responsive to treatment with RANKL (fault detection rate-corrected *p* < 0.05) and had fold change greater than 1.5; Lastly, using Gene Ontology annotations the genes relating to osteoclasts were filtered and selected as target genes. For each of the target genes, the number of positive hits among the 10 algorithms was grayscale-coded as a confidence measure of *in silico* prediction.

### 4.5. Transfection of the Inhibitor and Mimic of miR-221-3p, miR-222-3p and c-Src siRNA

Cells were treated with the inhibitor and mimicking agent of miR-221-3p and miR-222-3p, as well as siRNA specific to c-Src (Life Technologies). The selected target sequences were: 5′-AGC UAC AUU GUC UGC UGG GUU UC-3′ (inhibitor and mimic of miR-221-3p); 5′-AGC UAC AUC UGG CUA CUG GGU-3′ (inhibitor and mimic of miR-222-3p); and 5′-GGA AGA ACC CAU UUA CAU U-3′ (c-Src siRNA). As a nonspecific control, mirVana™ miRNA Inhibitor Negative Control #1 (5′-UUA CGU CGU CGC GUC GUU AU-3′), mirVana™ miRNA Mimic Negative Control #1 (5′-UAA CGA CGC GAC GAC GUA A-3′), and a negative siRNA (5′-UGU ACU GCU UAC GAU UCG G-3′) (Life Technologies) were employed. Using Opti-MEM I medium and Lipofectamine RNAiMAX (Life Technologies), cells were transiently transfected with siRNA for c-Src or control. The medium was replaced by regular culture medium after 12 h, and the silencing efficiency was evaluated with immunoblotting or quantitative PCR 48–72 h after transfection.

### 4.6. Quantitative Real-Time PCR

Using an RNeasy Plus mini kit (Qiagen, Germantown, MD, USA) and a high capacity cDNA reverse transcription kit (Applied Biosystems, Carlsbad, CA, USA), total RNA was extracted and reverse transcription was performed. Quantitative real-time PCR was then conducted using ABI 7500 with Power SYBR green PCR master mix kits (Applied Biosystems). We evaluated miRNA levels of miR-27b-3p, miR-125b-5p, miR-146a-5p, miR-182-5p, miR-221-3p, and miR-222-3p (Table 1), as well as mRNA levels of NFATc1, TRAP, cathepsin K, Dcstamp, and c-Src using primers listed in Table 2. SnoRNA202 (for miRNA) and GAPDH (for mRNA) were used for internal control.

**Table 1:** Targeted miRNA sequences.

miRNA	Targeted miRNA Sequences
27b-3p	5′-UUCACAGUGGCUAAGUUCUGC-3′
125b-5p	5′-UCCCUGAGACCCUAACUUGUGA-3′
146a-5p	5′-UGAGAACUGAAUUCCAUGGGUU-3′
182-5p	5′-UUUGGCAAUGGUAGAACUCACACCG-3′
221-3p	5′-AGCUACAUUGUCUGCUGGGUUUC-3′
222-3p	5′-AGCUACAUCUGGCUACUGGGU-3′
SnoRNA202	5′-GCTGTACTGACTTGATGAAAGTACTTTTGAACCCTTTTCCATCTGATG-3′

**Table 2:** Real-time PCR primers for mRNAs.

Gene	Forward Primer	Backward Primer
*c-Src*	5′-TCCTTTGGGATTCTGCTGAC-3′	5′-TGTGGCTCAGTGGACGTAAA-3′
*cat K*	5′-CAGCTTCCCCAAGATGTGAT-3′	5′-AGCACCAACGAGAGGAGAAA-3′
*Dcstamp*	5′-AAAACCCTTGGGCTGTTCTT-3′	5′-AATCATGGACGACTCCTTGG-3′
*NFATc1*	5′-GGTGCTGTCTGGCCATAACT-3′	5′-GCGGAAAGGTGGTATCTCAA-3′
*TRAP*	5′-TCCTGGCTCAAAAAGCAGTT-3′	5′-ACATAGCCCACACCGTTCTC-3′
*GAPDH*	5′-TGCACCACCAACTGCTTAG-3′	5′-GGATGCAGGGATGATGTTC-3′

### 4.7. Western Immunoblotting

Using a radioimmunoprecipitation assay buffer that contained protease inhibitors (Santa Cruz Biotechnology, Santa Cruz, CA, USA) as well as phosphatase inhibitors (Calbiochem, Billerica, MA, USA), cells were lysed for harvesting proteins. Isolated proteins were size-fractionated using 10%–12% sodium dodecyl sulfate gels and blotted to a polyvinylidene fluoride membrane (Millipore, Billerica, MA, USA). Incubation with primary antibodies was conducted for 1 h, followed by 45 min incubation with secondary antibodies conjugated with horseradish peroxidase (Cell Signaling, Danvers, MA, USA). Primary antibodies in this study included NFATc1 and cathepsin K (Santa Cruz Biotechnology), TRAP (Abcam, Cambridge, MA, USA), c-Src (Cell Signaling), and β-actin (Sigma, St. Louis, MO, USA). Protein levels were detected using a SuperSignal west femto maximum sensitivity substrate (Thermo Scientific, Waltham, MA, USA) and quantified with a luminescent image analyzer (LAS-3000, Fuji Film, Tokyo, Japan).

### 4.8. Live Cell Imaging for c-Src Activity Using Fluorescence Resonance Energy Transfer (FRET)

The activity of c-Src in response to the inhibitor of miR-222-3p was visualized via FRET imaging using a c-Src biosensor consisting of cyan fluorescent protein (CFP) and yellow fluorescent protein (YFP). The biosensors were transfected into RAW264.7 cells using a Neon transfection system (Life Technologies). Time-lapse images of c-Src FRET activity were collected every 2 min using a fluorescence microscope (Nikon, Tokyo, Japan). The selected wavelength was: CFP excitation at 438 ± 24 nm (center wavelength ± bandwidth), CFP emission at 483 ± 32 nm, and YFP emission at 542 ± 27 nm. Using NIS-Elements software (Nikon), the level of c-Src activity was determined as an emission ratio of CFP/YFP for individual cells and presented with the color bar.

### 4.9. TRAP Staining and Bond Resorption Assay

For TRAP staining, RAW264.7 cells were transfected with non-specific control siRNA or Src siRNA and cultured for 4 days in a 96-well plate (0.5 × 10^4^ cells/well) in the presence and absence of 50 ng/mL RANKL. TRAP staining was conducted using an acid phosphatase leukocyte kit (Sigma). In this study, TRAP-positive cells were defined as those that contained three or more nuclei. For a bone resorption assay, RAW264.7 cells were plated at 2.5 × 10^3^ cells/well using a bone resorption assay plate (Cosmo Bio, Tokyo, Japan) and cultured with 50 ng/mL RANKL for 5 days. Cells were then removed from the plate, and the pit area generated by osteoclast activity was determined using an optical microscope (TS100, Nikon, Tokyo, Japan).

### 4.10. Statistical Analysis

Data were expressed as mean ± S.D., based on three or four-independent experiments. ANOVA followed by *post hoc* tests was employed for evaluating statistical significance among multiple samples. For the mRNA microarray, a Benjamini–Hochberg correction was applied to determine the false detection rate. Statistical significance was evaluated at *p* < 0.05. The single and double asterisks indicate *p* < 0.05 and *p* < 0.01, respectively. Signal intensities in immunoblotting were determined using Image J (National Institutes of Health, Bethesda, MD, USA).

## 5. Conclusions

This study revealed that miR-222-3p suppresses RANKL-driven osteoclastogenesis. The observed suppression was mediated by c-Src, since inhibition of miR-222-3p elevated expression of c-Src and a partial silencing of c-Src reduced expression of the osteoclast marker genes such as TRAP and cathepsin K. The role of miRNAs has not been fully understood in bone-related metabolic diseases and cancer. The results of this study might contribute to developing a novel therapeutic strategy for prevention of bone loss in many skeletal diseases.
